# Intrapulmonary intra-arterial malposition of a port catheter—an exceptionally rare complication

**DOI:** 10.1007/s10354-025-01115-6

**Published:** 2025-11-12

**Authors:** Anton Busau, Edin Ahmic, Paul Swatek, Iurii Mykoliuk, Andrej Roj, Alfred Maier, Joerg Lindenmann

**Affiliations:** Division of Thoracic and Hyperbaric Surgery, Department of Surgery, Medical University of Graz, Graz, Austria

**Keywords:** Unusual complication of the portimplantation, Thoracic surgery, Intrapulmonary location of the port system, Port-a-Cath, Pulmonary segmental artery

## Abstract

Although port system implantations have been established as standardized surgical procedures, they may be associated with significant risks and complications. Therefore, both the planning and the correct execution of the implantation require a high degree of accuracy. To the best of our knowledge, the present case is the first report presenting a potentially life-threatening intrapulmonary intra-arterial malposition of a port catheter. A 66-year-old female patient was admitted to our division due to a port-a-catheter malposition. CT images showed an intrapulmonary localization of the port catheter, located in an apical pulmonary segmental artery in the right upper lobe. Urgent explantation of the port-a-cath was performed without complication, and the patient left our division on the fourth postoperative day. This extremely complicated case demonstrates the importance of accurate intra- and postoperative radiological imaging for rapid and distinct detection of perioperative complications after surgical implantation of port systems.

## Introduction

Numerous port complications have been described in the literature. Basically, they can be divided into intraoperative, early, and late complications. Intraoperative complications include bleeding, hematoma, pleural injury, pneumo- or hemothorax, cardiac arrhythmias, pericardial tamponade, and injury or irritation of the brachial plexus.

Infections; secondary bleeding or hematoma; and dislocation or disconnection of the port system with catheter leakage, catheter rupture, or perforation represent early postoperative complications. Late complications include catheter occlusion and catheter perforation (pinch-off syndrome), dislocation, disconnection, or migration [1, 2].

However intra-arterial placement of the port system has been still reported to have an estimated risk of between 1.1% and 3.7% [3, 4]. Port systems, as intracorporeal accesses with a significantly reduced risk of infection (0.8%–12.2% vs. 2.5%–38%) [5–8] compared to percutaneously placed catheters, represent a safe and permanent option for accessing the central venous system. However, the occurrence of complications depends on various factors, including the surgeon’s surgical experience and anatomical peculiarities.

This case report describes a serious and potentially life-threatening complication that has not previously been documented in the literature: intrapulmonary malposition of a port-a-cath with the catheter entering a pulmonary segmental artery of the right upper lobe closely located to the lung hilus.

## Case report

**Fig. 1:**
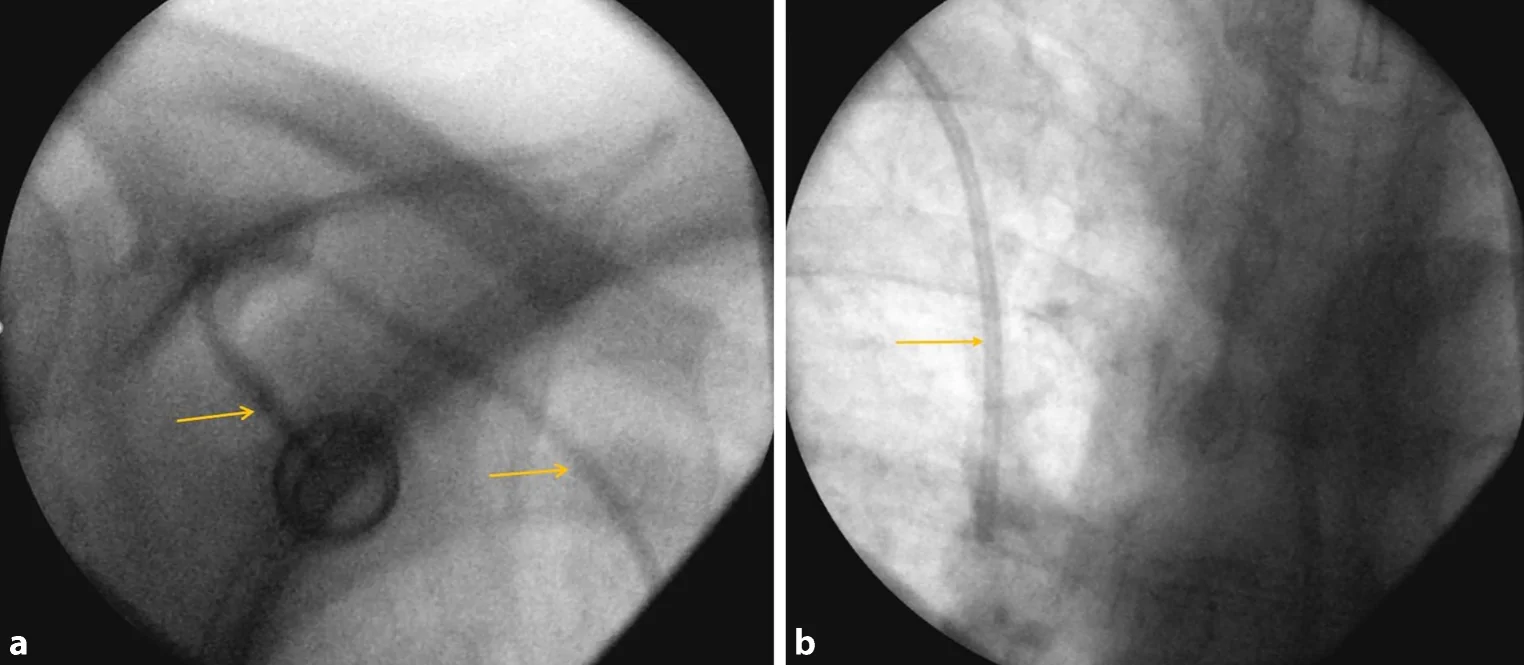
**a,** **b** The incorrectly obtained intraoperative radiological images

In a peripheral hospital, a 66-year-old female patient (162 cm, 65 kg) underwent right-sided port implantation for chemotherapy following breast cancer surgery. Because of intraoperative technical difficulties, the duration of this extremely prolonged surgical procedure was 4 h, during which two intraoperative images (Fig. 1a, b) were taken.

**Fig. 2:**
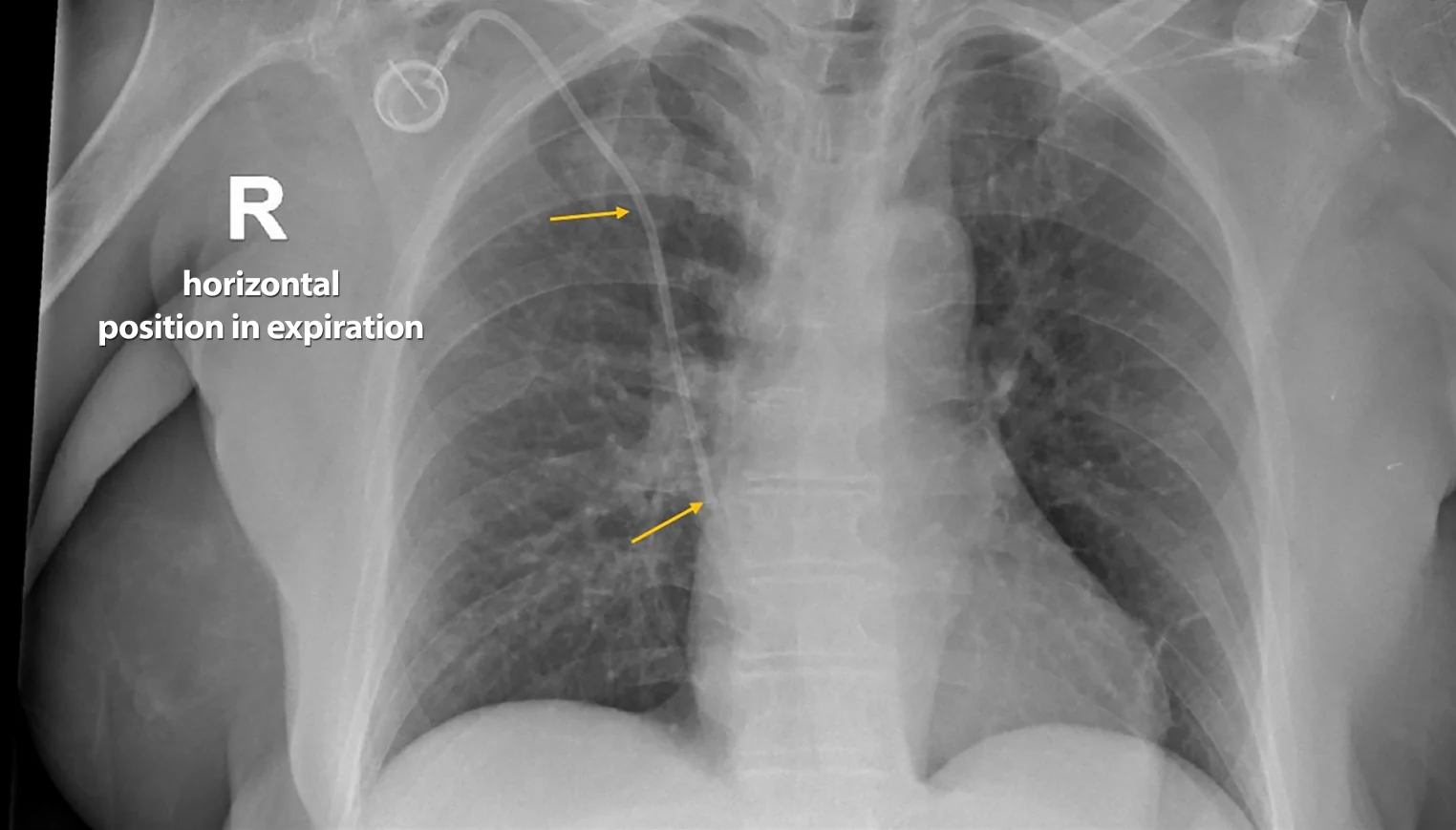
The postoperative chest X-ray, which already showed the intrapulmonary malposition of the Port-a-Cath catheter

**Fig. 3:**
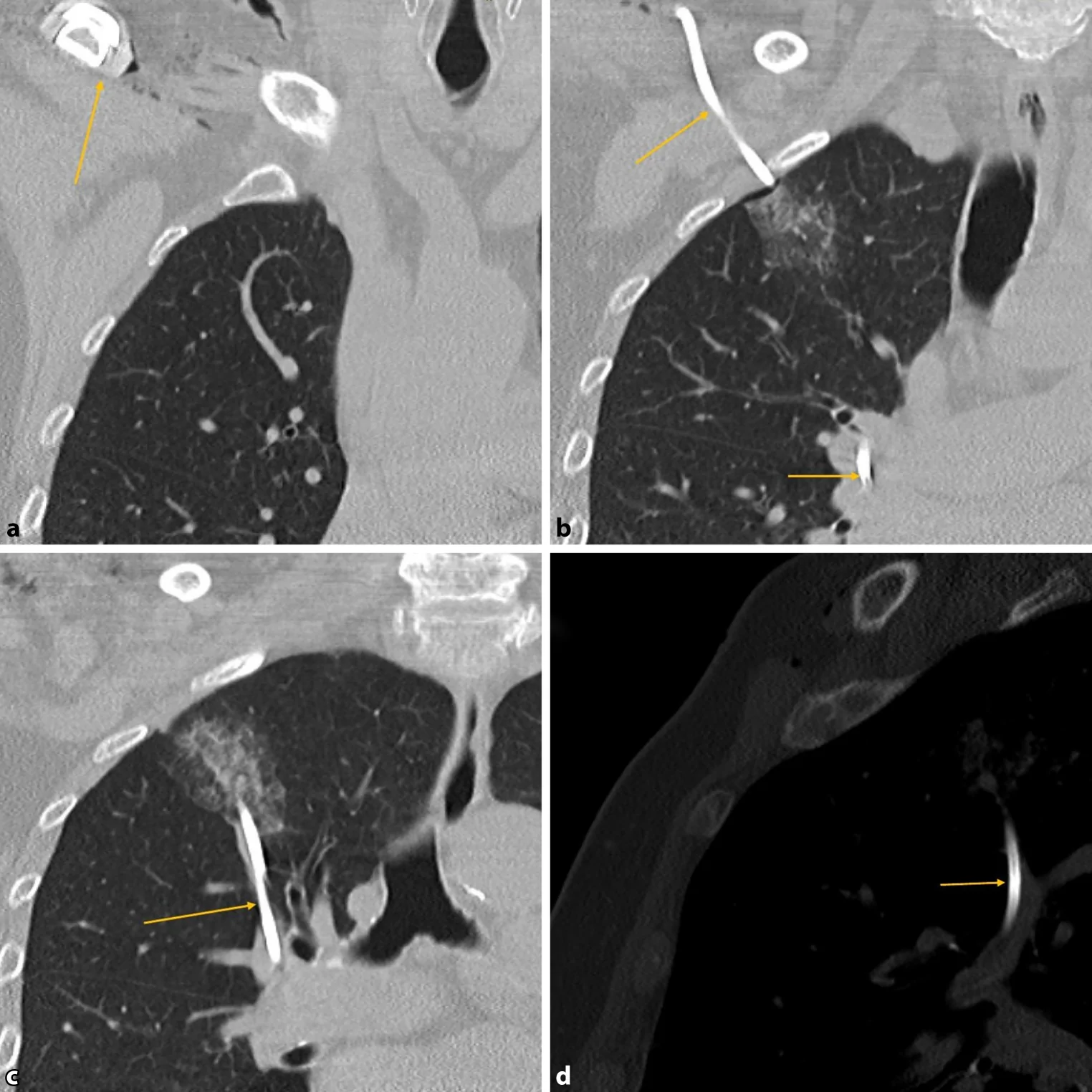
The CT images (**a**–**d**) clearly confirmed the intrapulmonary location of the port system, with the catheter positioned intravascularly in an apical segmental artery of the right upper lobe

After port implantation, a postoperative chest X‑ray was performed (Fig. 2), which already showed the intrapulmonary malposition of the port-a-cath. A computed tomography (CT) scan of the chest with contrast medium was then performed. The CT images (Fig. 3a–d) clearly confirmed the intrapulmonary location of the port system, with the catheter positioned intravascularly in an apical pulmonary segmental artery of the right upper lobe. Active vascular or pulmonary hemorrhage could not be confirmed at that time, nor could the presence of pneumo- or hemothorax.

After contacting our department, the patient was admitted for urgent surgical revision. Upon arrival the patient presented in a respiratory and hemodynamically stable condition (Glasgow Coma Scale 15/15, body temperature 36.6 °C, blood pressure 105/60 mm Hg, pulse rate 81/min, respiratory rate 16/min, SpO_2_ 99%, pH 7.39, RBC 3.67 10^12^/L, Hb 11.7 g/dL, Hct 32.7%, creatinine 0.57 mg/dL).

Immediately after admission to the hospital, the patient, who was circulatory stable, non-intubated, and spontaneously breathing, underwent urgent surgical treatment for this complication. After double-lumen intubation and in a left lateral thoracic position, a right-sided anterolateral mini-thoracotomy was performed, followed by initial evacuation of 500 ml of hemothorax. After dissecting the right pulmonary hilum and exposing the pulmonary segmental arteries supplying the right upper lobe of the lung, the affected pulmonary segmental artery 1b was clearly identified. After gradually withdrawing the port catheter from the arterial lumen, the segmental artery was dissected without complication using a clip and ligature. After slowly removing the port catheter from the lung parenchyma, careful monitoring of the bleeding in the lung parenchyma was performed, and the intraparenchymal catheter channel was adequately closed with parenchymal sutures.

**Fig. 4:**
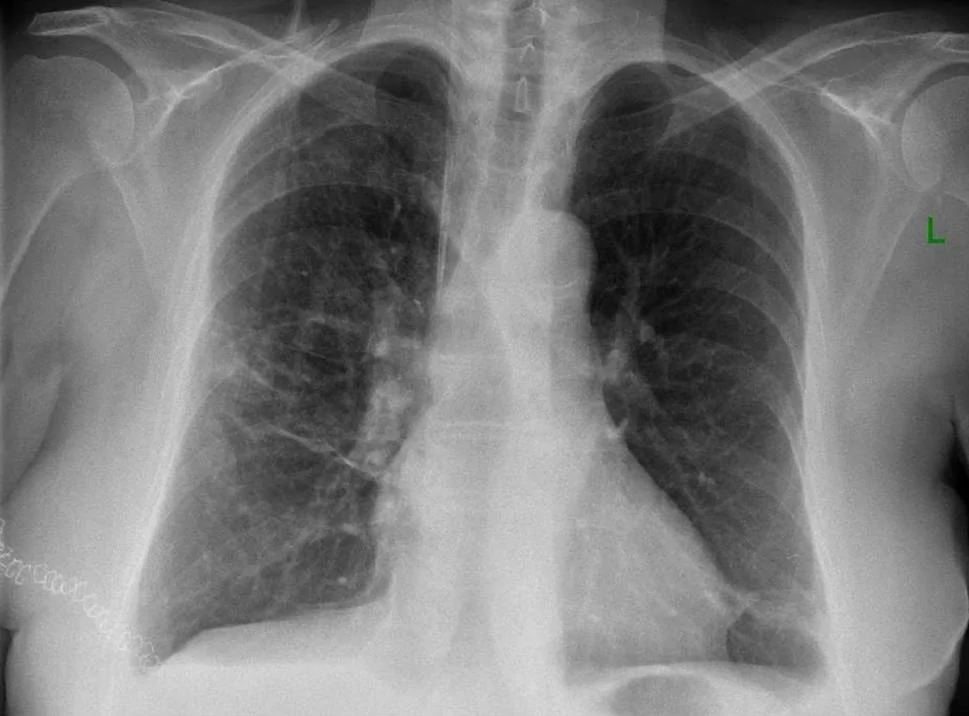
Final chest X‑ray

The postoperative course was uneventful, and the chest drain was removed on the third postoperative day. The terminatory chest X‑ray showed no evidence of secondary parenchymal bleeding, pneumothorax, or hemothorax (Fig. 4). The patient was discharged for outpatient care on the fourth postoperative day.

## Discussion

Intra-pulmonary placement of a port system with the catheter tube entering a pulmonary segmental artery represents a very rare and extremely unusual complication.

In the course of a recent literature review, similar complications were detected, such as rupture of an iatrogenic pseudoaneurysm of the pulmonary artery after implantation of a Swan–Ganz catheter or port system [9, 10] and dislocation of a catheter fragment into a pulmonary segmental artery [11]. To the best of our knowledge, the present case is the first case report worldwide presenting this potentially life-threatening complication.

In our opinion, two anomalies are particularly noteworthy in this case. On the one hand, the above-average prolonged duration of the operation (4 h), and on the other hand, the incorrectly obtained intraoperative radiological images (Fig. 1a, b). Ultimately, these two factors contributed significantly to the incorrect implantation of this port system.

In the current case, the right-sided cephalic vein could be identified, but the catheter could not be advanced further within this narrow vessel. In order to overcome this obstacle, ultrasound-guided puncture of the corresponding subclavian vein was performed. During this prolonged intraoperative procedure, a leak in the port catheter was detected. As a consequence, the port catheter had been replaced using the Seldinger technique. However, it is a matter of debate which fact was causative for this malposition of the port catheter. On the one hand, incorrect interpretation of the sonographic images displaying the subclavian vein could be possible. On the other hand, we cannot rule out incorrect performance of the Seldinger procedure by replacing the port catheter.

One can assume that the main surgeon might not be fully familiar with this surgical procedure because a second surgeon had joined during surgery to speed up the operation. As we do not know anything about the surgeon’s experience, the incorrect interpretation of the intraoperative radiological images might be a strong hint for a lack in experience in this kind of operations. However, finally, based on both the correct interpretation of the postoperative chest X‑ray (Fig. 2) and the CT scan of the chest (Fig. 3a–d), this surgical complication was clearly identified and addressed. The rapid transfer of the patient to our department and the immediate surgical revision allowed this complication to be corrected quickly and uneventfully, thus preventing further considerable harm to the patient.

In this case could be of course an option of treatment in the first line an interventional radiological treatment (embolisation of the affected pulmonary artery). However we decided to perform the open approach by a small thoracotomy in order to avoid any further needless complication in this exceptional case.

In order to prevent such complications, pre- and/or intraoperative Doppler sonography of the affected vessels (cephalic vein, subclavian vein) could be helpful [12]. Furthermore, meaningful intraoperative radiological control represents a pivotal surgical component, both for verifying the correct localization of the port system and the catheter and for responding to and correcting deviations from the surgical norm in a timely manner.

In this context, the appropriate position of the implanted port catheter should be confirmed during surgery using X‑ray fluoroscopy, which gives an excellent overview and allows for quick and detailed assessment. The tip of the port catheter should be located about two vertebral bodies below the level of the main carina. To rule out thrombosis of the subclavian veins, preoperative vascular ultrasound or CT with contrast medium should be performed to evaluate vascular patency. When these radiological examinations are conducted preoperatively, the operation time can be shortened, and intraoperative vascular complications can be prevented in advance.

In the current case, vascular obstruction of the subclavian vein could be ruled out by the CT scan conducted before the transfer to our department. However, the patient had undergone an uneventful left-sided mastectomy several years ago, but postoperative radiation therapy had never been administered. Considering these facts, one should not have expected any intraoperative complications for the subsequent surgical implantation of the right-sided port catheter.

However, the earliest possible detection and prompt treatment of such misplacements are still essential to prevent further complications.
